# Being as an iceberg: hypertensive treatment adherence experiences in southeast of Iran

**DOI:** 10.3402/gha.v8.28814

**Published:** 2015-09-21

**Authors:** Nahid Dehghan Nayeri, Mahlagha Dehghan, Sedigheh Iranmanesh

**Affiliations:** 1 Nursing and Midwifery Care Research Center, School of Nursing and Midwifery, Tehran University of Medical Sciences, Tehran, Iran; 2 Department of Medical Surgical Nursing, School of Nursing and Midwifery, Kerman University of Medical Sciences, Kerman, Iran

**Keywords:** hypertension, treatment adherence, qualitative content analysis, Iran, lived experience

## Abstract

**Background:**

Treatment adherence is often an important issue in the management of hypertension. Deep understanding of adherence behavior as well as its influential factors can expand knowledge about treatment adherence among hypertensives.

**Objective:**

The aim of this study was to explore patients, their families, and healthcare providers’ experiences about hypertension treatment adherence in southeast of Iran.

**Design:**

A qualitative study was conducted to explore the experience of patients, family members, and healthcare providers (*n*=18) by using a conventional content analysis. The purposive sampling method was used. Data were collected through semi-structured and deep interviews.

**Results:**

Data analysis showed that hypertensive treatment adherence in an Iranian context is like an iceberg with two subthemes. The first subtheme relates to the upper and clear part of this iceberg and it consists of two categories, including 1) healthy and 2) unhealthy regimens. The second subtheme associates with under-water and unanticipated part and it consists of four categories, including 1) the nature of disease and treatment, 2) the individual resources, 3) the healthcare organization, and 4) the socio-cultural environment.

**Conclusions:**

The treatment adherence features emerged in this study can be useful in designing and developing context-based hypertension interventions. Further qualitative and quantitative studies with a closer collaboration between the social, natural, and medical sciences in other Iranian populations are needed to confirm the findings.

High blood pressure is an important global public health threat as it is the most common risk factor for cardiovascular, cerebrovascular, and kidney diseases (1, 2). The prevalence of hypertension varies in different regions; however, it was estimated that 26.4% of adults had hypertension in 2000 and its prevalence has been predicted to increase about 60% (1.56 billion) by 2025 (3). Insufficient control is the most common factor for a hypertension crisis (4). The lack of adherence to a recommended treatment regimen is often the main reason for poorly controlled hypertensive patients (5). According to the World Health Organization (WHO), approximately 75% of hypertensive patients are not sufficiently controlled because of poor adherence to the treatment regimen (6). Non-adherence associates with the worsening of disease, increased mortality, frequent hospitalization, and high rates of morbidity, and it leads to substantial avoidable healthcare costs (7, 8).

According to a review of literature, it has been shown that various factors in different countries with different socio-cultural environments influence hypertensive treatment adherence (9, 10). Patient beliefs and knowledge about hypertension (9, 11–15), patient–provider relationship (10, 16, 17), social support (17, 18), depression (12), preference for alternative and traditional treatment (10, 19), self-efficacy (17), and motivation (20) were some of the factors that directly or indirectly, positively or negatively influenced hypertensive treatment adherence. In addition, Marshall et al. (21), in their systematic review of qualitative research, reported that most of the participants believed that their hypertension was caused by stress and it was improved by stress relief. In addition, they intentionally reduced or stopped their treatment. They reported that patients with hypertension disliked treatment and were afraid of medication side effects and addiction (21). They also found that forgetfulness, being busy, cost of treatment regimen including healthy food, and appointments prevented adherence to a treatment regimen. These findings were the same in different countries (US, UK, Brazil, Sweden, Canada, New Zealand, Denmark, Finland, Ghana, Iran, Israel, The Netherlands, South Korea, Spain, Tanzania, and Thailand) (21).

## Context

Iran is a large country located in southwest Asia and the Middle East with a population of about 76 million (22). Iranian people are believed to be religious with the majority being Muslim (23). These Iranian characteristics might cause variations in the adherence to the anti-hypertensive treatment regimen. A meta-analysis in Iran showed that the prevalence of hypertension between the ages of 30 and 55 is 23% (4). The analysis also showed that 50% of people older than 55 years were affected by hypertension (4). According to Javadi et al. (24), only 5% of Iranian hypertensive patients follow their prescribed regimen and have controlled blood pressure. Mohammadi et al. (25), in their qualitative study, explained that ‘non-compliance’, ‘lack of knowledge’, ‘lack of effective caring relationship’, and ‘necessity of partnership’ were the main interrelated concepts that influence high blood pressure control among Iranian patients. Literature review showed that few studies focused on hypertensive treatment adherence among the Iranian population. However, these quantitative studies focused on hypertensive medication adherence and did not focus on hypertensive treatment adherence or factors affecting adherence (26, 27). Therefore, it is necessary to have a comprehensive understanding about adherence concept in hypertensive patients to determine suitable strategies and improve hypertensive adherence (28). Thus, a qualitative approach could help understand the meaning of hypertensive treatment adherence. In addition, understanding facilitators and barriers affecting treatment adherence in hypertensive patients might allow researchers and healthcare providers to design and develop effective interventions. Moreover, a cultural-based understanding may help healthcare policy makers to establish effective context-based interventions. The aim of this study was to describe experiences of patients, family members, and healthcare providers about hypertension treatment adherence and to explore factors affecting adherence in their lived experiences.

## Materials and methods

### Study design and setting

This study was a conventional, qualitative content analysis with a descriptive–explorative approach. Content analysis is a qualitative method of analyzing written, verbal, or visual communication messages. The aim of content analysis research is to attain a condensed and broad description of the phenomenon. According to Thyme et al. (29) and Graneheim and Lundman (30), content analysis can be performed with various degrees of interpretation. They stated that in each text, there are manifest messages vs. latent messages although both messages require interpretations which may vary in depth and level of abstraction (29, 30). In this study, we attempted to analyze the latent content as well as manifest content. People of different ethnicities live in Iran (Azeri in the northwest, Kurdish in the west, Arabs in the south and southwest, Fars in the center, Turkmen in the northeast, and Baluch in the east). They have different cultures, lifestyles, and socio-economic status (22). This study was conducted in Kerman. Kerman is the largest city in southeast of Iran, with a population of more than 722,000. People with different religious beliefs live in Kerman province including Shia Muslims, Zoroastrians, and Sheikhiyya sect and each have specific religious rituals. In addition, Kerman is the living place and location of various nomadic groups who have not been influenced by urban culture yet. In general, Kerman culture has been based on two important factors including the specific climate and natural environment and history of hard battles. Therefore, the cultural and ecological characteristics of this city resulted in cardiovascular departments of the educational hospitals admitting people with different lifestyles.

### Sampling, participant, and data collection

As in qualitative research, no absolute rules determine the estimated number of participants, sampling continued until data were saturated and no new information was extracted. In the present study, the saturation was achieved after interviewing 18 participants. Purposive sampling was used to select participants. The participants were selected and contacted by the cardiovascular departments or physician offices. Seven participants were interviewed in hospitals affiliated by Kerman University of Medical science (KUMS). Two interviews were done in Razi Nursing and Midwifery school. The remaining interviews were conducted in participants’ homes with prearranged appointments. To capture rich and diverse information, individuals (patients, family members, nurses, and physicians) with different and rich experience about the research concept were invited to do the interviews. In addition, individuals with different characteristics such as age, role, and work experience were chosen by the second researcher to provide a wide range of information. In total, 10 hypertensive patients, four family members of hypertensive patients (two of the family members had no relationship with patients who participated in the study and the other two were son and wife of different selected patients), two nurses, and two physicians were interviewed. The age of the patients varied between 38 and 74 years. Three patients had sufficient controlled blood pressure, meaning that their blood pressure dropped to less than 140/90 mmHg (31). Half of the patients were females. The duration of being affected by hypertension varied between 2 and 25 years. Half of the patients who participated in the study were retired. Nurses and physicians had 5–12 years of experience of caring for hypertensive patients (Table 1). Just one patient avoided participating in the study because of an unwillingness to speak up and voice recording. The recruitment was based on some inclusion criteria. Patients were required to have at least one-year experience of living with hypertension. The following characteristics were also required of family members: 1) aged 18 years or older and 2) at least 2 years of experience of living with a hypertensive patient. Nurses and physicians needed to have at least 2 years of experience of caring for hypertensive patients. All participants were required to speak Persian.

**Table 1:** Characteristics of the participants (*n*=18)

Participant	Gender (male/female)	Age (years)	Experience of illness/living with hypertensives/work (years)	Job/relative to patient
Patients	5, 5	38–74	2–25	Pensioner: 5 Employee: 1 Teacher: 1 Housewife: 2 University Lecturer: 1
Family member	1, 3	20–40	1–25	Daughter Wife Sister Son
Healthcare team	2, 2	27–45	5–12	Nurse: 2 Physician: 2

Semi-structured, in-depth, face-to-face individual interviews were conducted by one of the researchers (MD). Some of the questions that elicit participants’ experiences about hypertensive treatment adherence are shown in Table 2. Interviews lasted 25–60 min. The interviews were audio taped and then written out verbatim. The sampling took place from August 2013 to March 2014.

**Table 2:** Example of questions

Participant	Question
Patients	1) How much is your blood pressure? Is it controlled?, 2) In your daily living, how do you adhere to hypertensive treatment regimen?, 3) How about physician's recommendation about diet and medication?, 4) What about exercise?, 5) In your experience which factors affect your adherence?
Family members	1) Does your patient adhere to his/her treatment regimen?, 2) What did you do to make your patients adhered to his/her regimen?, 3) In your daily living with a hypertensive patient which factors facilitate her/his treatment adherence?, 4) What were the barriers?
Healthcare providers	1) Do your hypertensive patients adhere to their treatment regimen?, 2) In your daily care of hypertensive patients which factors affect their treatment adherence?, 3) Which factors were related to healthcare system and healthcare providers?

### Ethical consideration

KUMS approved this project. The Ethics Committee of KUMS confirmed all processes and procedures used in the study (Ethical code: k/93/580). After approval, some oral information was given to the participants including the goals and objectives of the study, the confidentiality and anonymity of the data, and that they were free to withdraw from the study at any time. The first researcher then invited each participant and verbal consent was given individually.

### Data analysis

To perform conventional qualitative content analysis, the following concepts were considered important: unit of analysis, meaning unit, condensation, code, category, and theme (30). The qualitative content analysis is based on the unit of analysis. According to Graneheim and Lundman (30), unit of analysis is those interviews that are large enough to be considered as a whole and small enough to keep in mind as a context for the meaning unit during the analysis process. In our study, each interview considered a unit of analysis. After determining unit of analysis, the text was divided into meaning units. Each meaning unit consists of words, sentences, or paragraphs containing aspects related to each other through their content and context. In our example, the part of text describing get the habit to hypertension constituted one meaning unit and the part explaining patient description about medication adverse effects constituted another (Table 3). In the next step, we condensed the meaning units, while still preserving the core. The condensed meaning units were then labeled with a code and sub-categories were created. The next step was to create categories that are the core feature of qualitative content analysis. A category is a group of codes that are similar in a manifest level. In our example, one category describes the healthy regimen and another states the nature of disease and treatment (Table 4). A theme is a recurrent thread of underlying meaning running through codes and categories; it can be seen as an expression of the latent meaning of a text. A theme can be divided into sub-themes on different levels. In our example, one subtheme describes adherence to different aspects of hypertensive treatment regimen and it was labeled by ‘The tip of the hypertensive treatment adherence iceberg’ (Table 4). A theme is an interpretative level of underlying meanings of condensed meaning units, codes, and categories (30). Although the analysis process was systematic, it was a back-and-forth movement between the whole and parts of the text. Table 3 gives an overview of the analysis process executed on each text, and Table 4 gives an overview of all subcategories, categories, and subthemes. The analysis process lasted from August 2013 to October 2014.

**Table 3:** Example of qualitative content analysis process

Meaning unit	Condensed meaning unit	Code	Subcategory	Category	Subtheme	Theme
When I take Amlopres, my blood pressure fall to 12 and it makes me not to be fine, i.e. when my blood pressure is 16, I feel better than when it is 12. (Patient No. 8)	By improving blood pressure the symptoms were manifested	Appearance of symptoms by blood pressure improvement	Get the habit to hypertension	Being connected to the nature of disease and treatment	The floating platform of the hypertensive treatment adherence iceberg	Hypertensive treatment adherence as an iceberg
Since I have done regular checkups in previous years my hypertension was detected, otherwise I had no hypertensive symptoms such as dizziness or being uncomfortable. (Patient No. 9)	No symptoms were seen during increasing of blood pressure	Lack of symptoms during hypertension	Hypertension as a silent illness			
I feel that my body has not adapted to Losartan and Metoral. At first, I tried to adhere to Doctor's prescription to take half of the pills, and then I took one pill in the morning and one at night, but with no effect. (Patient No. 8)	The prescribed medication and frequent changes of medication had no effect on the patient	No hypertension control despite frequent changes of medication	Hypertension as a resistant illness			
She took the medication for some days, the medication was not consistent with her body and she coughed severely, so severely that it seemed her tonsils were coming out. It was very bad. (Patient's daughter, No. 1)	The patient coughed severely while taking medication	Coughing after taking medicine	Medication adverse effect			
The patient does not like to take multiple medications. For example, they argued that why do they have to take three medications to reduce blood pressure of 14/9! (Physician No. 12)	The patients were reluctant to take multiple medications for mild high blood pressure	No tendency to take multiple medications for mild hypertension	Need to consuming more drugs			

**Table 4:** Theme, sub-themes, categories, and subcategories

Theme	Sub-theme	Category	Sub-category
Hypertensive treatment adherence as an iceberg	The tip of the hypertensive treatment adherence iceberg	Being connected to the healthy regimen	Diet regimen
			Medication regimen
			Monitoring
			Exercise
		Being connected to the unhealthy regimen	Smoking
			Stress
	The floating platform of the hypertensive treatment adherence iceberg	Being connected to the nature of disease and treatment	Get the habit to hypertension
			Hypertension as a silent illness
			Hypertension as a resistant illness
			Medication adverse affect
			Need to consuming more drugs
		Being connected to the individual resources	Beliefs, attitudes, and knowledge
			Values and desires
			Emotional and mental concerns
			Experiences and lessons
			Forgetting and being busy
		Being connected to healthcare organization	The role of government and the Health Ministry
			The role of doctors and the medical team
		Being connected to the socio-cultural environment	Insistence offering culture
			Non-knowledgeable counseling
			Traditional and herbal treatments
			Media
			The context of the family

### Trustworthiness

Trustworthiness is a criterion which constitutes rigor in qualitative researches. Four issues are normally used to describe various aspects of trustworthiness: credibility, confirmability, dependability, and transferability (30, 32). Several techniques were used to enhance trustworthiness of the following study. Peer checking has been done by the second researcher's supervisors (the first and third researchers). Through frequent sessions between the second researcher and the supervisors, the study's progress and process was reported and discussed. Member checking was completed with some of the participants for validation of interpreted findings (codes and categories). Some of the faculty members checked the encoding process and accessing categories (external checks). In addition, a clear and detailed description of culture, context, selection, and characteristics of participants, data collection, and process of analysis were provided.

## Findings

### Theme, sub-themes, and categories

According to the participants’ experiences, adherence to hypertensive treatment in the Iranian context seems to be like an iceberg. An iceberg has obvious and hidden layers. Getting deeper to the underwater portion of the iceberg is like revealing hidden factors that impact patient's ‘adherence to hypertensive treatment’. These rooted factors are difficult to judge by looking at the tip of the iceberg. This indicates that ‘hypertensive treatment adherence’ is a small manifestation of a larger problem. The theme consists of two subthemes: ‘The tip of hypertensive treatment adherence iceberg’ and ‘The floating platform of hypertensive treatment adherence iceberg’ (Table 4).

## The tip of hypertensive treatment adherence iceberg

According to the participants’ explanations, being connected to ‘healthy regimen’ and ‘unhealthy regimen’ were the main concepts that a hypertensive patient must comply with to capture high blood pressure control.

### Being connected to healthy regimen

Adherence to hypertensive treatment was considered as a healthy behavior such that the patient has to obey the healthcare recommendation regarding suitable diet, taking medication, monitoring blood pressure and factors affecting it, and increasing physical activity.

One diabetic hypertensive patient stated, ‘I wrote the time of taking the pills on their packets according to doctor's prescription and took them regularly’.

The Daughter of a hypertensive man who was also a nurse stated, ‘For the first time, the doctor told my father to change his diet into a moderate regimen i.e., the salt should not be omitted rather he should take it less than before. He also advised frequent walking, with no medication. My father closely followed his recommendation’.

One patient with two years of experience of hypertension stated, ‘I always checked my blood pressure once a week in hospital or in health clinic. I was more careful’.

### Being connected to unhealthy regimen

According to participants’ experiences, adhering to hypertensive treatment is needed to stop or reduce some unhealthy behaviors, such as smoking, opium consumption, and stressful events.

One 73-year-old patient stated, ‘The doctor told me: do not smoke cigarette. At that time, I usually smoked 6 or 7 cigarettes. What I mean is that I smoked one cigarette at ten o'clock, another one after lunch, and still smoked another one in the afternoon at 4 o'clock. I did not smoke all the time. The doctor ordered me not to smoke so I stop smoking cigarettes. I did not use the smokes that the Kerman people use (he meant opium)’.

One 58-year-old patient stated, ‘I always visits neurologist. I was nervous; I was getting better with their pills. The doctor always gave me pills, but he increased the pills this year and I don't know the reason. I told the doctor: I am not nervous, I am ok, I am not angry, I do not quarrel with my children but my wife believes that I am angry’.

## The floating platform of hypertensive treatment adherence iceberg

The adherence to hypertensive treatment has been influenced by several contexts directly or indirectly. According to the participants’ explanations, being connected to ‘the nature of disease and treatment’, ‘the individual resources’, ‘the healthcare organization’, and ‘the socio-cultural environment’ were the main contexts which influence the adherence to hypertensive treatment.

### Being connected to the nature of disease and treatment

Participants explained that hypertension has some specific features. Some of the participants had no special signs and symptoms when their blood pressure increased. Some felt better with high blood pressure than normal blood pressure. Some had resistant high blood pressure such that medication and lifestyle modification did not improve their illness. Medication side effects and the need to take more drugs (different types with different divided doses) were other features for the treatment of hypertension that caused participants to no longer adhere to their treatment regimen.

One patient with five year hypertension stated, ‘When I take Amlopres, my blood pressure falls to 12 and it makes me not to be fine, i.e., when my blood pressure is 16, I feel better than when it is 12’.

The daughter of a 48-year-old patient stated, ‘She took the medication for some days, the medication was not consistent with her body and she coughed severely, so severely that it seemed her tonsils were coming out. It was very bad’.

A 38-year-old patient, an instructor at a University, who was aware of her hypertension and its treatment, stated, ‘At the beginning, I did not want to believe in my disease, I tried to correct my diet, I was very active, I did exercises but I considered my diet and because the minimum was high, it was difficult to control it. Since I could not deal with it, I started taking medicine’.

### Being connected to the individual resources

‘Beliefs, attitudes, and knowledge’, ‘values and desires’, ‘emotional and mental concerns’, ‘experiences and lessons’, and ‘forgetting and being busy’ were the main personal factors that positively or negatively influenced participants’ adherence behaviors. Generally, the majority of participants had a negative attitude toward the disease, treatment, doctor, and medication; for example, some of them believed that their body had this ability to recover itself and consulting doctors for such a disease is not cost-effective. Others believed that their hypertension is inherent and could not be treated. Some emphasized that their hypertension directly depends on nervousness and relief as they relax. The participants also believed that chemical drugs had more cons than pros and they did not want to use drugs for slightly high blood pressure. Some of the participants stated that their religious values had a positive effect on adherence. They mentioned that their body is a loan from God, and they should carefully pay attention to what they eat and how to care of this loan. In addition, they tried to have more patience in stressful situations and to make themselves relaxed by religious activities such as being always connected to God. Some participants believed that pleasures had a negative effect on their adherence. They stated that adhering to treatment regimen was against their pleasure principle and if they adhere to their treatment regimen, they would not enjoy their life. The participants explained how emotional and mental concerns affect their adherence behavior. They stated that having stressful, serious, strict, irritable, depressive, and obsessive personality made it more difficult for them to adhere to all aspects of the treatment regimen. Some participants mentioned that experiencing some of the side effects of high blood pressure such as blurred vision and numbness of the tongue caused them to adhere more to their treatment regimen. Also, being busy (i.e. working or taking care of children and family) and forgetting were other personal factors that negatively influenced participants’ adherence behaviors.

One patient with six years of hypertension stated, ‘Since my childhood days, I refused to visit doctor. In my childhood, when I felt so ill, my father forced me to visit the doctor. I do not like visiting the doctor at all’.

A 74-year-old hypertensive man stated, ‘What affects me the most is my nervousness. For example, if someone speaks badly, that is if the dialogue is face to face, I become extremely nervous but I do not hit, curse or struggle with them’

The son of a hypertensive couple stated, ‘God has given me a trust that is my body. It is very important. I am not important rather the trust given to me is very important so I try not to eat such food’.

A doctor with 10 years experience stated, ‘It is difficult to remove fast foods from the diet because people are highly involved with their daily lives and do not have enough time for cooking. People such as employees, students and even doctors have to eat fast foods due to lack of time’.

A 69-year-old patient who has experienced hypertension for 25 years stated, ‘When my tongue was paralyzed, I became afraid and was more careful. That day lasted as if it is one year for me. I was very anxious and unhappy because I could not speak well. I was getting mad. I hope that nobody faces such situation’.

A 72-year-old patient stated, ‘I sometimes took my medicines. When I was nervous or I had guests, or I was changing my house, I forgot to take my medicine, but when my children were at my house, they gave me the medicine’.

### Being connected to the healthcare organization

According to the participants’ experiences, healthcare organization had some positive and negative effects on hypertensive treatment adherence. They mentioned that the key roles of ‘government and the health ministry’ were to provide more accessible care, suitable medicines, and follow-up systems. The majority of participants complained that the offices of the physicians’ were often crowded and they had to waste so much time in their offices. In addition, physicians and medical team commitments, skills, and behaviors had special effect on patients’ adherence to treatment.

One Doctor with 12 years experience stated, ‘Lack of compound drugs in our country is an important issue especially for hypertensive regimens such that several drugs with low doses have better effects than one drug with high dose. This can be a reason why patients do not adhere to medical regimens’.

A nurse with seven years experience stated, ‘There was one patient with high level of education. He was a professor in a University (Mathematical PhD). He asked his doctor to prescribe pills instead of recommending walking and lifestyle modification. He told his doctor: I cannot focus on whatever you asked me to do. I have a busy mind. But the doctor told him: that is a pity; you want to take hypertensive pills. His blood pressure was 14/9 but it bothered him because it was constant. After several months, he was hospitalized due to severe M.I (myocardial infarction) and he complained to the doctor why he did not prescribe pills for him.

### Being connected to the socio-cultural environment

The participants stated that ‘insistence offering culture’, ‘non-knowledgeable counseling’, ‘traditional and herbal treatments’, ‘media’, and ‘the context of family’ were the most important socio-cultural environments that affect adherence behaviors. The first three factors had more negative impact on adherence to hypertensive treatment, while the last two factors had more positive effects. The participants explained that they find it difficult to resist, when their relatives or friends offer them unhealthy foods such as pizza or fast foods. Some participants emphasized that they followed the recommendations of non-specialized persons on their illness. Using traditional and herbal treatments made some participants not to adhere to their regimen consistently. Some participants mentioned that information in the media helped them to know more about their illness and treatment. The majority of the participants explained that a supportive family atmosphere had a positive impact on adhering to treatment regimen including eating safe foods, exercising, recalling medication usage, and following physician appointments.

A 47-year-old patient stated, ‘Sometimes, it happens that my colleagues buy sandwiches and we have to eat as well. If we do not eat it, they will insist on eating the sandwich, sometimes they buy sweets and we have to eat them’.

A patient with two-year hypertension stated, ‘My wife does not allow me to eat salty foods. I wish to eat salty foods but she avoids me. My children tell their mother not to allow me to eat salty foods as well’.

A 67-year-old patient with hypertensive complications such as renal failure stated, ‘My mother or old relatives told me to take herbal medicines such as hollyhock. It was not very effective on me. I did what others told me and I went to grocery and bought what others told me such as green tea or Mecca tea in order to treat my blood pressure but they had no effect on me’.

One of the nurses who participated in the study stated her experience about the effect of TV programs on adherence of a patient:

One of my patients controlled his creatine based on TV health programs. In the TV programs, it was said for example, if blood creatine is high, the patient should reduce taking Losartan and Captopril and inform the doctor if she/he is affected by renal disease. In TV program, it was said that renal disease can be diagnosed by a blood test called creatine. The patient did the test accordingly and his creatine was one tenth or two tenth higher than the normal range so he stopped taking his pills without referring to his doctor. Then he was referred to the emergency with a severe high blood pressure.

## Discussion

The aim of this study was to explore lived experience of hypertensive patients, their families, and healthcare providers regarding treatment adherence and its influential factors in Kerman, Iran. According to the participants’ experiences, individual, socio-cultural, and healthcare organizational factors had important influences on different aspects of treatment adherence. Diet and medication regimens, monitoring, exercise, smoking and stress are the most important issues in treatment adherence. The results also revealed that adhering to these treatment issues was influenced by the nature of disease and its related treatment.

According to the international guidelines (33), to manage hypertension, patients must adhere to medication regimen and modify their lifestyle. Modifications of diet, increasing physical activity, stopping smoking as well as reducing stress are recommended to be adhered (33–36). Reducing alcohol drinks is another important unhealthy behavior which is considered to be adhered among hypertensive patients (37). However, participants in the present study did not experience alcohol consumption. The majority of Iranians are Muslims. According to Quran–Muslims’ holy book–drinking alcohol is forbidden [2:219]. In addition, given that Iran has a theocracy government and that alcohol consumption is prohibited by the religion, the public accessibility to alcohol is not easy. This was in contrast with an earlier study conducted in China (36). In addition, the present study revealed that adherence to regularly monitoring blood pressure is another important issue for managing hypertension. This finding is also recommended by The Seventh Report of the Joint National Committee on Prevention, Detection, Evaluation, and Treatment of High Blood Pressure and earlier studies (33, 36). It should be mentioned that although diet, medication, exercise, stress reduction, and stopping smoking are considered as different aspects of treatment adherence in the present study, these comprehensive results are due to using different participants. On the other hand, all of these adherence aspects were not experienced by patients. The patients had focused less on monitoring and stopping smoking. Jolles et al. (8) and Mohammadi et al. (25) confirmed that patients did not have complete and comprehensive knowledge about hypertension and its treatment and that there are differences between healthcare providers and patients’ knowledge and attitude about hypertension.

According to the participants’ experiences, the nature of hypertension and its treatment made patients not to adhere to treatment regimens. According to clinical guidelines, hypertension is an asymptomatic disease with a long-term threat. The body will adapt to high blood pressure so that the complications will gradually manifest with no special symptoms. This is why it is called a silent killer (33, 38). Moreover, the text revealed that resistant high blood pressure needs to be treated with different types and different dosages of medications, as well as medications’ side effects, which was another challenging dimension. Thus, all these complex and confusing conditions lead patients not to adhere to their treatment adherence regimen appropriately. These findings were consistent with earlier studies (9–11, 14, 16, 19, 21, 38, 39) that were conducted in different countries with various socio-cultural contexts. Therefore, these factors are suggested to be fundamental and cross-culturally embedded dimensions of hypertension adherence behaviors.

Participants experienced that patient’ beliefs and knowledge about hypertension, depression, fear of addiction, forgetfulness, and being busy were some individual barriers of adhering behaviors, which is consistent with the experiences of participants in previous researches (9–12, 14–16, 19–21, 40). According to the findings of this study, the majority of patients believed that stress was the main cause of hypertension which is in agreement with the results of earlier studies (21, 38). According to a systematic review, acute stress is probably not a risk factor for hypertension while chronic stress and non-adaptive response to stress are more likely causes of sustained elevation of blood pressure (41). In addition, another systematic review reported that the available stress-reduction techniques used for at least 6 months appeared to reduce blood pressure in patients with hypertension, but they should be interpreted with caution because of major methodological limitations. According to this systematic review, the benefit of specific stress-reduction techniques in hypertensive patients remains unproven (42). Therefore, it is clear that patients in Iran or in other countries are not well informed about the nature and cause of high blood pressure and related treatment. However, according to the Iranian hypertensives, the more a person wants to enjoy his or her life, the less he or she adheres to a treatment regimen. In addition, being connected to Islamic religious rules about avoiding overeating and oversleeping made some participants change their lifestyles based on these rules. According to Islam, people are free to get enjoyment from all aspects of their life but this freedom will be limited and blamed whenever they harm their material and spiritual life (Quran 7:32). Moreover, Kretchy et al. (43) in their quantitative study reported that spirituality significantly related with medication non-adherence while religion had no effect on adherence. This is in contrast with the findings of further studies. Iran is a religious-based context and people are required to follow religious rules; these beliefs could contribute to patients’ changing their lifestyle based on Islamic beliefs and values. However, further quantitative studies are needed to be done in order to support qualitative findings.

The present study showed that physicians and medical team's commitments, skills, and behaviors had special motivational effect on patients’ adherence behaviors. Similarly, earlier researches (9, 10, 12, 15, 17, 38, 40, 44–46) reported that trust in a doctor and a mutual patient–provider relationship facilitated adherence to the treatment. Based on the findings, other Iranian healthcare organizational factors that affect treatment adherence included lack of accessible care, suitable medicines, and follow-up systems. According to a recent report by WHO, by 2005, the total number of physicians per 1,000 population in Iran was 0.89, that is, less than one physician per 1,000 population (47). Therefore, physicians’ offices often are crowded and they dedicate a little time to visit each patient. In addition, there is no suitable and comprehensive follow-up system in Iran. A family physician program has recently been started (since 2014) yet the majority of people are not serviced by this system. Lack of combined medication (two or three kinds of medicine in one drug) in Iran is another problem. Unfortunately, an international sanction (since 2006) made this problem worse. These barriers were not addressed by earlier qualitative studies conducted among hypertensive patients in different contexts (9–12, 14, 16, 17, 20, 21, 39, 40). In studies that followed, the above barriers (except physicians’ offices crowding) were not experienced by patients, but perceived by healthcare providers. It could be related to the fact that healthcare providers compared to the patients are more engaged with organizational deficiencies and are more aware of newly developed combined medicines.

The results also revealed that socio-cultural factors play an important role in patients’ adherence to hypertension treatment. In Iran, especially in Kerman province, people culturally used to insist on preparing and taking foods to parties and meetings. In addition, refusing offers from other means disrespect and annoys the host. This may cause patients to eat unhealthy foods or to overeat. This cultural habit seems to be to somewhat similar to Malaysian culture. According to Shima et al., most hypertensive participants had difficulty in controlling their diet due to the widespread availability of food in Malaysia. They continued by saying that a common reason for not following a low-salt and low-fat diet was that they were not supported by family and peers to resist salty and fatty foods (46).

The text revealed that applying non-specialist persons’ recommendations about disease and treatment is another challenging factor that is mostly experienced by low-educated patients compared to those with a high level of education. In Iran, lots of people who are not experts on health issues and diseases use to talk and give suggestions about health and disease-related issues. According to the literature, the prevalence of self-medication among Iranians varies between 35.7 and 91% (48–50). The main causes of self-medication are previous illness experience, ease of access to most of medications, feeling that the illness is not important, and advice from friends and families (48–50). Therefore, these socio-cultural contexts cause people to apply each other's recommendations easily. According to Shima et al. (46), Indian patients were more influenced by people surrounding them, especially their families (spouse, mother-in-law) and peers (neighbors, friends), in their decision-making on medication adherence in comparison with Malayan and Chinese patients. Patients also preferred to use traditional and herbal treatments as they believed that these medicines have less complications compared to chemical drugs. Social support including information provided by media and a supportive family encouraged patients to adhere to their regimen. These findings were supported by earlier studies (9, 12, 17, 46).

As with most studies, our study had some limitations. Even though the participants in the current study did not experience alcohol consumption, it is considered a health issue in Iran. However, it is not exactly clear whether participants consumed alcohol drinks or they did not want to talk about it as talking about alcohol consumption in a religious context is unpleasant.

In conclusion, the results of this study supported previous knowledge about hypertensive adherence behavior and also created new insights toward Iranian contextual factors that influence this topic. The results showed that hypertensive treatment adherence in Iran consisted of adhering to medication, diet, exercise, monitoring, and reducing smoking and stress. All aspects of treatment adherence were affected by different factors including individual, socio-cultural environment, healthcare organization, and nature of disease and treatment. Different values and desires, insistence offering culture, non-knowledgeable counseling, and effect of sanctions on health organization were some specific findings. To develop hypertensives’ adherence behaviors, it is important to understand patients, family, and healthcare providers’ perceptions. A comprehensive context-based understanding of the phenomenon may assist healthcare providers and policy makers to design suitable patient-centered, family-centered, and society-centered interventions. The results of following study may provide useful information for development of such programs. The present study can provide guidance to the public about the causes of hypertension and the lifestyle changes necessary to adequately cure hypertension. Further qualitative and quantitative studies with a closer collaboration between the social, natural, and medical sciences in other Iranian populations are needed to confirm the findings.
